# Prejudice and misconceptions about tuberculosis and HIV in rural and urban communities in Ethiopia: a challenge for the TB/HIV control program

**DOI:** 10.1186/1471-2458-10-400

**Published:** 2010-07-06

**Authors:** Amare Deribew, Gemeda Abebe, Ludwig Apers, Chali Jira, Markos Tesfaye, Jafar Shifa, Alemseged Abdisa, Kifle Woldemichael, Fetene Deribie, Mesele Bezabih, Abraham Aseffa, Robert Colebunders

**Affiliations:** 1 Department of Epidemiology, Jimma University, Jimma, Ethiopia; 2 Department of Epidemiology and Social Medicine, University of Antwerp, Antwerp, Belgium; 3 Department of Medical Laboratory Sciences and Pathology, Jimma University, Jimma, Ethiopia; 4 Department of Clinical Sciences, Institute of Tropical Medicine, Antwerp, Belgium; 5 Department of Health Service Management, Jimma University, Jimma, Ethiopia; 6 Department of Psychiatry, Jimma University, Jimma, Ethiopia; 7 Department of Internal Medicine, Jimma University, Jimma, Ethiopia; 8 Armaur Hansen Research Institute, Addis Ababa, Ethiopia

## Abstract

**Background:**

In Ethiopia, where HIV and tuberculosis (TB) are very common, little is known about the prejudice and misconceptions of rural communities towards People living with HIV/AIDS (PLHA) and TB.

**Methods:**

We conducted a cross sectional study in Gilgel Gibe Field Research area (GGFRA) in southwest Ethiopia to assess the prejudice and misconceptions of rural and urban communities towards PLHA and TB. The study population consisted of 862 randomly selected adults in GGFRA. Data were collected by trained personnel using a pretested structured questionnaire. To triangulate the findings, 8 focus group discussions among women and men were done.

**Results:**

Of the 862 selected study participants, 750(87%) accepted to be interviewed. The mean age of the respondents was 31.2 (SD ± 11.0). Of the total interviewed individuals, 58% of them were females. More than half of the respondents did not know the possibility of transmission of HIV from a mother to a child or by breast feeding. For fear of contagion of HIV, most people do not want to eat, drink, and share utensils or clothes with a person living with HIV/AIDS. A higher proportion of females [OR = 1.5, (95% CI: 1.0, 2.2)], non-literate individuals [OR = 2.3, (95%CI: 1.4, 3.6)], rural residents [OR = 3.8, (95%CI: 2.2, 6.6)], and individuals who had poor knowledge of HIV/AIDS [OR = 2.8, (95%CI: 1.8, 2.2)] were more likely to have high prejudice towards PLHA than respectively males, literates, urban residents and individuals with good knowledge. Exposure to cold air was implicated as a major cause of TB. Literates had a much better knowledge about the cause and methods of transmission and prevention of TB than non-literates. More than half of the individuals (56%) had high prejudice towards a patient with TB. A larger proportion of females [OR = 1.3, (95% CI: 1.0, 1.9)] and non-literate individuals [OR = 1.4, (95% CI: 1.1, 2.0)] had high prejudice towards patients with TB than males and literate individuals.

**Conclusion:**

TB/HIV control programs in collaboration with other partners should invest more in social mobilization and education of the communities to rectify the widespread prejudice and misconceptions.

## Background

The high burden of TB/HIV co-infection and the emergence of multi-drug resistance TB (MDR-TB) are a growing concern for developing countries particularly for Africa[1]. Most (79%) of the TB/HIV co-infected patients reside in Africa[1].

In 2008, Ethiopia ranked 7^th ^and 3^rd ^in terms of the total number of incident cases of pulmonary and extra pulmonary TB among the 22 highly affected countries in the world[1,2]. Tuberculosis is the first cause of hospitalization and the third cause of adult mortality in the country. The numbers of TB cases are increasing every year and a total of 1,166,863 TB cases have been identified and registered for Directly Observed Short Course Therapy (DOTS) in the last 10 years[3]. Ethiopia is also one of the Sub-Saharan African countries heavily affected by HIV/AIDS with a point prevalence of 2.2%. Currently, there are an estimated 1.3 million PLHA in Ethiopia. The rate of TB HIV co-infection is high in Ethiopia, ranging from 25% to 57%, in different regions of the country[4-8].

Behavioral factors such as poor health care seeking behavior [9-13], poor adherence to anti-retroviral[14-16] and anti-TB [17-19] treatments, stigma and discrimination towards patients with TB [20-22] and HIV[23-25] are major challenges for TB/HIV control programs in low income countries. Empowerment of the community at grass root level through effective advocacy, communication and social mobilization[26] is a crucial step to achieve the TB and HIV related millennium development goals [27].

To design effective behavioral intervention strategies, the level of prejudice and misconceptions of the community towards PLHA and patients with TB should be evaluated. Several studies about stigma and misconceptions among patients with TB [20,21,28-35] and PLHA [23,24,36,37] have been done previously. In Ethiopia, where more than 85% of the population reside in rural areas, very little is known about this subject. We conducted a quantitative and qualitative community based survey in a predominantly rural area of southwest Ethiopia to address this knowledge gap.

## Methods

From February to March, 2009, a cross sectional community based survey was conducted in GGFRA located 260 Km southwest of Addis Ababa, the capital of Ethiopia. The GGFRA was established in 2005 to serve as a research center and field attachment site of Jimma University. The research center comprises of eight rural and two urban *Kebeles *(lowest administration unit in Ethiopia) which are located around the reservoir of Gilgel Gibe hydroelectric dam. The total population of the field research center is 50156 with 10,859 households.

### Study Population

The source population consisted of adults older than 14 years of age who lived in the 10 Kebeles of the GGFRA. From the source population, a total of 862 adults were proposed to be included for the quantitative survey by considering the following assumptions: prevalence of prejudice towards PLHA of 50%, 95%CI, margin of error of 3.5% and a non-response rate of 10%. The total sample size was proportionally distributed to the ten study Kebeles. In each Kebele, households were selected by simple random sampling using the unique household number. In a household, one adult person was selected randomly for the interview. To get the target study participants, households were visited repeatedly. If the respondent was not found in three visits, the next household was included to replace another respondent. Individuals younger than 15 years old or temporary residents were excluded from the study. To triangulate the findings of the quantitative survey, eight (4 among women and 4 among men) focus group discussions (FGD) were conducted. For each FGD, 8-10 individuals who were supposed to have adequate information were selected in consultation with the chairpersons of the study Kebeles.

### Measurements

Quantitative data were collected by trained personnel who completed grade 12. They used pretested structured questionnaire. The questionnaire included socio-demographic variables, knowledge about HIV/AIDS (15 items), prejudice or stigma towards PLHA (10 items), knowledge concerning TB (5 items), prejudice towards a patient with TB (14 items) and health care seeking behavior of the community for TB. The knowledge and prejudice questions for HIV [38,39] and TB[20,38] were adopted from published questionnaires. The internal consistencies of the HIV and TB prejudice scales were 0.71 and 0.83 respectively. Knowledge on the transmission of HIV was measured by 8 questions with yes/no responses: Is HIV transmitted by a mosquito/fly?, by eating raw beef (a common tradition in Ethiopia) prepared by a person who lives with HIV?, by eating together with a patients with HIV?, from a pregnant woman to her child?, by breast feeding?, by eating an uncooked egg laid by a chicken which swallowed a used condom?, by unprotected sexual practice?, and through an injury by unsterile sharp objects? Prejudice about HIV was evaluated by 10 questions: should a person living with HIV/AIDS be isolated?; are you willing to share a meal with a person living with HIV/AIDS?, to buy food from a hotel owned by a person living with HIV/AIDS?, to care for an HIV positive female relative?, to care for an HIV infected male relative?, to disclose the sero-status of HIV infected household member?, to allow an HIV infected family member to take Anti-retroviral Treatment (ART)?; do you allow a family member to marry without an HIV counseling and testing?; do you believe an HIV positive student can continue his education?; do you believe an HIV infected teacher should pursue his/her work? The TB prejudice scale included the beliefs of the respondents on the isolation of TB patients; disclosure of TB to others; perception about the social, sexual and marital problems of TB patients. Based on these questions, the degree of prejudice on HIV and TB was scored. An answer consistent with prejudice towards HIV or TB was scored with one point. An answer not consistent with prejudice towards HIV or TB was scored as zero points. A total prejudice score for HIV or TB was created by summing the scores of all questions. The HIV prejudice score ranged from 0 to 10, with the higher the score, the greater the degree of prejudice towards HIV. Individuals who had a prejudice score of equal to or greater than the mean score of the study population were categorized as having high prejudice towards HIV or TB. On the other hand, individuals who scored a prejudice score below the mean were categorized as having low prejudice towards HIV or TB. Since the prejudice scores of HIV and TB were normally distributed, the means were used to classify the study population as having high or low prejudice.

Similar scoring was done for the knowledge questions concerning HIV and TB. Qualitative data were collected by experienced public health and health education experts using FGD guides which consisted of stigma, knowledge, attitude, and health seeking behaviors of the community for TB.

### Data analysis

Data were double entered using Epi-data version 3.1(Epi-data, Norway, 2006). For analysis, the data were exported to SPSS version 16.0 statistical software (SPSS Inc. Chicago, 2007). Descriptive analysis was done to measure knowledge, stigma and health care seeking behavior. Pearson's Chi-square was used to assess the association between socio-demographic variables and knowledge with prejudice. To control for the effect of confounding variables, stepwise logistic regression was done. Variables which had a statistically significant association (P < 0.05) in the Pearson' Chi-square test were included in the final logistic regression model. All FGD interviews were transcribed by public health and health education experts immediately after the interview. The transcribed data were commented by the investigators. After several readings, key categories & themes were identified. The data were interpreted and presented verbatim.

### Ethical considerations

The proposal was approved by the ethical review committees of Jimma University, Armaur Hansen research institute and the Institute of Tropical Medicine, Belgium. Written consent was obtained from the study participants.

## Results

### Socio-demographic characteristics of the study population

Of the 862 selected study participants, 750(87%) accepted to be interviewed; 112(13%) declined. Majority (70%) of those who refused to accept the interview were males. The mean age of the respondents was 31.2 (SD ± 11.0). Of the total interviewed individuals, 85.2%, 75% and 58% were Muslims, married and females respectively. Six hundred and forty five (86%) of the study participants were Oromo by ethnicity. The median monthly income of the individuals was 400 Ethiopian Birr (34 USD)/per month (Table 1).

**Table 1:** Socio-demographic characteristics of the study population (n = 750), southwest Ethiopia

Variable	Number (%)
**Sex**	
Male	315(42)
Female	435(58)
**Literacy status**	
Literate	290(38.3)
Non-literate	460(61.7)
**Age in years**	
15-24	216(28.8)
25-34	250(33.3)
35-44	164(21.9)
=>45	120(16.0)
**Mean age(SD)**	31.2(±11.0)
**Religion**	
Muslim	639(85.2)
Orthodox Christian	95(12.7)
Protestant	16(2.1)
**Ethnicity**	
Oromo	645(86.0)
Yem	42(5.6)
Amhara	32(4.3)
Gurage	17(2.3)
Keffa	5(0.7)
Dawro	3(0.4)
Tigre	2(0.2)
Other	4(0.5)
**Occupation**	
Farmer	372(49.6)
Housewives	184(24.5)
Government employee	69(9.2)
Day laborers	25(3.3)
Student	41(5.5)
Merchant	47(6.3)
No job	12(1.6)
**Marital status**	
Married	563(75.1)
Single	125(16.7)
Divorced	24(3.2)
Widowed	38(5.1)
**Monthly Income**	
<400 Ethiopian Birr(<34USD)	410(58.0)
=>400 Birr(=>34 USD)	297(42)
**Area of residence**	
Rural	531(70.8)
Urban	219(29.1)

### Knowledge and perception towards HIV/AIDS

Almost all of the study participants (97.5%) had heard of HIV/AIDS. More than half of the respondents did not know the possibility of transmission of HIV from a mother to a child or by breast milk. A larger proportion of non-literate individuals were more likely to have misconceptions about transmission of HIV than literates (Table 2).

**Table 2:** Perception of the study participants concerning the cause of HIV/AIDS (n = 731), southwest Ethiopia

Indicators of conception	sex		P-value	Literacy status		P-value
				
	Male No(%)	Female No(%)		Non-literate No(%)	Literate No(%)	
**Vector can transmit HIV**			0.02			0.001
Yes	20(6.4)	46(11.0)		53(12.0)	13(4.5)	
No	294(93.6)	371(89.0)		389(88.0)	276(95.5)	
**Eating uncooked egg laid by a chicken that swallowed a used condom can transmit HIV**			0.23			0.15
Yes	70(22.3)	78(18.7)		82(18.6)	66(22.8)	
NO	244(77.7)	339(81.3)		360(81.4)	223(77.2)	
**Eating raw meat prepared by a person who lives with HIV can transmit HIV**			0.83			0.08
Yes	62(19.7)	85(20.4)		98(22.2)	49(17.0)	
No	252(80.3)	332(79.6)		344(77.8)	240(83.0)	
**Eating together with an HIV infected person can transmit HIV**			0.04			0.004
Yes	23(7.3)	49(11.8)		55(12.4)	17(5.9)	
NO	291(92.7)	368(82.2)		387(87.6)	272(94.1)	
**Unsterile syringes and sharp objects can transmit HIV**			0.15			0.08
Yes	298(94.9)	383(91.8)		406(91.9)	275(95.2)	
NO	16(5.1)	34(8.2)		36(8.1)	14(4.8)	
**Breast feeding can transmit HIV to the baby**			0.59			0.005
Yes	138(43.9)	175(42.0)		171(38.7)	142(49.1)	
NO	176(56.1)	242(58.0)		271(61.3)	147(50.9)	
**HIV can be transmitted from mother to child during pregnancy and labor**			0.73			0.001
Yes	138(43.9)	178(42.7)		168(38.0)	148(51.2)	
NO	176(56.1)	239(57.3)		274(62.0)	141(48.8)	
**Unprotected sex can transmit HIV**			0.09			0.004
Yes	263(83.8)	329(78.9)		343(77.6)	249(86.2)	
NO	51(16.2)	88(21.1)		99(22.4)	40(13.8)	

The majority of the FGD participants believed that HIV/AIDS is a punishment from God for unacceptable human sexual behavior.

"*These days, extramarital sexual practices are becoming more common. As a punishment for this sexual infidelity, Rabbi/God has given the human being an incurable disease called AIDS*". A 50 years old Muslim in a rural Kebele

The majority of the male FGD participants described that unprotected sexual practices with multiple partners such as polygamous marriage in their village could play a major role for the transmission of HIV/AIDS. On the other hand, the majority of the female participants believed that the mosquito that transmits malaria and flies could transmit the virus. The role of flies as a transmission agent for HIV was exemplified by a 35 years old woman:

"*Flies can transmit AIDS from an HIV infected person's wound to a healthy person. Fortunately, in our village, there are no patients with HIV who could transmit the disease to us*."

### Prejudice towards HIV/AIDS

One hundred and seventy six (24%) participants had high prejudices towards PLHA. A higher proportion of females [OR = 1.5, (95% CI: 1.0, 2.2)], non-literate individuals [OR = 2.3, (95%CI: 1.4, 3.6)], rural residents [OR = 3.8, (95%CI: 2.2, 6.6)], individuals who had poor knowledge on HIV/AIDS [OR = 2.8, (95%CI: 1.8, 2.2)] and individuals with lower income [OR = 1.7,(95%CI:1.1,2.6)] had high prejudice towards PLHA than respectively males, literates, urban residents, individuals who had good knowledge and a higher income (Table 3).

**Table 3:** Predictors of prejudice towards people living with HIV/AIDS (n = 728), southwest Ethiopia

Variables	Prejudice		Crude OR (95% CI)	Adjusted OR (95%CI)
			
	High	Low		
**Sex, no (%)**				
Male	63(20.2)	249(79.8)	1	1
Female	113(27.2)	303(72.8)	1.5(1.0,2.1)	1.5(1.0, 2.2)
**Literacy status, no (%)**				
Literate	34(11.8)	253(88.2)	1	1
Non-literate	142(32.2)	299(67.8)	3.5(2.3,5.3)	2.3(1.4, 3.6)
**Age in years, no (%)**				*
15-24	38(17.9)	174(82.1)	1	
25-30	65(26.6)	179(73.4)	1.6(1.0, 2.6)	
35-44	31(19.3)	130(80.7)	1.1(0.6, 1.8)	
>=45	42(37.8)	69(62.2)	2.8(1.6, 4.6)	
**Occupation, no (%)**				*
Government employee	4(5.9)	64(94.1)	1	
Farmer	105(28.8)	259(71.2)	6.5(2.3, 18.2)	
Housewives	53(30.3)	122(69.7)	6.9(2.4, 20.0)	
Day laborers	2(8.0)	23(92.0)	1.3(0.2, 8.1)	
Student	3(7.5)	37(92.5)	1.3(0.3, 6.1)	
Merchant	7(15.6)	38(84.4)	2.9(0.8, 10.7)	
Unemployed	2(18.2)	9(81.8)	3.5(0.5,22.3)	
**Monthly income in Birr, no (%)**				
<=400(<=34 USD)	123(31.0)	274(69.0)	2.4(1.6,3.6)	1.7(1.1, 2.6)
>400(>34 USD)	45(15.5)	246(84.5)	1	1
**Residence, no (%)**				
Rural	157(30.7)	355(69.3)	4.5(1.7,7.6)	3.8(2.2, 6.6)
Urban	19(8.8)	197(91.2)	1	1
**Knowledge on HIV, no (%)**				
Poor	136(31.2)	300(68.8))	2.8(1.8,4.2)	2.8(1.8,2.2)
Good	40(13.7)	252(86.3)	1	1

*Excluded in the final model

HIV/AIDS was a highly stigmatized disease in the community. Almost all of the FGD discussants described that people in their villages do not want to eat, drink, share utensils or clothes and participate in a social group such as "*Iddir*" with PLHA. *Iddir *(funeral insurance) is a community based organization whereby people contribute money or food regularly to support the family of a deceased individual. The major reason of stigma towards PLHA was the fear of transmission of the virus through direct contact. To substantiate this idea, a 42 years old woman said,

"*In fact, there is nobody who is infected with 'AIDS' in our village. If there is one, nobody will dare to visit his/her house, eat together, shake hands and exchange materials like clothes and utensils*."

The idea of isolation and discrimination of PLHA from social groups was illustrated by a 36 years old woman:

"*HIV infected person should not be a member of an Iddir. Nobody shakes hands with that person or touches the money contributed by him/her*."

Real example of discrimination against a person who was believed to have HIV/AIDS was discussed by the female FGD participants in one of the Kebeles.

"*In our village there was a man who was very emaciated and developed sores in his mouth. We used to run and hide when we saw him for fear of the transmission of HIV by hand shaking. Currently, his health is improved and most of us are embarrassed to see his eye*." A 36 years old woman at a rural Kebele

### Utilization of voluntary counseling and testing and condom

Three hundred and thirty nine (45%) and 512(70.2%) participants had heard of ART and voluntary counseling and testing. Of those who knew HIV counseling and testing, 212 (28%) were tested at least once. Six hundred and thirty four (84.5%) individuals have heard of condoms. In the 12 months prior to the survey, 521(69.5%) of the study participants had sexual intercourse, 27(5.2%) with more than one partner. Only 1.3% of individuals who had one sexual partner and 11% of those who had multiple sexual partners had utilized a condom at least once in the last one year.

### Knowledge and perception towards TB

A total of 708 (94.4%) participants had ever heard of TB. Literates had a much better knowledge about the cause, symptoms, and methods of transmission and prevention of TB than non- literate individuals (Table 4).

**Table 4:** Perception of the participants on the cause, method of transmission and prevention of Tuberculosis in southwest Ethiopia

Indicators of conception	Sex		P-value	Literacy status		P-value
				
	Male No (%)	Female No (%)		Non-literate No (%)	Literate No (%)	
**Ever heard of TB(n = 750)**			0.07			0.001
Yes	303(96.2))	405(93.1)		422(91.7)	286(98.6)	
No	12(3.8)	30(6.9)		38(8.3)	4(1.4)	
**Cause of TB (n = 708)**			0.6			0.001
Microorganism	147(48.5)	204(50.4)		170(40.3)	181(63.3)	
Not microorganism	156(51.5)	201(49.6)		252(59.7)	105(36.7)	
**Methods of Transmission of TB (n = 708)**						
Contaminated food and water	56(18.5)	71(17.5)	0.7	80(19.0)	47(16.4)	0.4
Through air during coughing and sneezing	276(91.1)	370(91.4)	0.9	371(87.9)	275(96.2)	0.001
Unsterile milk	25(8.3)	36(8.9)	0.8	21(5.0)	40(14.0)	0.001
Poor personal hygiene	21(6.9)	20(4.9)	0.6	20(4.9)	17(4.0)	0.2
**Symptoms of TB (n = 708)**						
Cough of 2 or more weeks	239(79.7)	315(77.8)	0.5	322(76.5)	232(81.7)	0.09
Fever	90(29.7)	135(33.3)	0.3	116(27.5)	109(38.1)	0.003
Weight loss/becoming thin	143(47.2)	158(39.0)	0.029	162(38.4)	139(48.6)	0.007
Hemoptysis	171(56.4)	219(54.0)	0.5)	222(52.6)	168(58.7)	0.5
Excessive night sweating	60(19.8)	61(15.1)	0.09	58(13.7)	63(22.0)	0.004
Chest pain	62(20.5)	60(14.8)	0.05	72(17.1)	50(17.5)	0.8
Shortness of breath	63(20.8)	53(13.1)	0.006	73(17.3)	43(15.0)	0.4
Poor appetite	63(20.8)	54(13.3)	0.02	54(12.8)	63(22.0)	0.002
**Prevention of TB (n = 708)**						
Cover mouth during coughing or sneezing	238(78.8)	306(76.1)	0.5	316(75.6)	228(79.7)	0.3
Proper disposal of sputum	180(59.6)	243(60.4)	0.6	229(54.8)	194(67.8)	0.002
Ventilation of houses	60(20.5)	63(15.7)	0.2	56(13.4)	69(24.1)	0.001

In the qualitative study, we identified different concepts of causation of TB. The majority of the FGD participants believed that TB was caused by exposure to *cold air*. A 43 years old man expressed the link between TB and cold air as:

"*People contract TB when they are exposed to cold air. The cold air, particularly which comes through open window will cause severe injury to the lung; cough gradually develops which ultimately becomes TB*."

The qualitative findings revealed other causes of TB such as alcohol, Khat (natural stimulant from Catha edulis plant) and exchange of drinking and eating utensils with a TB patient. It was believed that locally made strong alcohol called 'Katikala' and Khat could cause TB through their direct toxic effect on the lung. Participants believed that TB patients should have separate eating plates and drinking cups since the microorganism could not be removed from the plate or dish by washing alone.

### Prejudice towards TB

Three hundred and ninety nine (56%) individuals had high prejudice towards a patient with TB. A larger proportion of females and non-literate individuals had high prejudice than males and literate individuals (Table 5).

**Table 5:** Factors associated with prejudice towards a patient with tuberculosis (n = 708), southwest Ethiopia

Variables	Prejudice towards TB patient		Crude OR (95% CI)	Adjusted OR (95%CI)
			
	High	Low		
**Sex, no (%)**				
Male	156(51.5)	147(48.5)	1	1
Female	243(60.0)	162(40.0)	1.4(1.0,1.9)*	1.3(1.0, 1.9)*
**Literacy status, no (%)**				
Literate	146(51.0)	140(49.0)	1	1
Non-literate	253(60.0)	169(40.0)	1.4(1.0,1.9)*	1.4 (1.1, 2.0)*
**Age in years, no (%)**				*
15-24	120(59.1)	83(40.9)	0.8(0.5,1.3)	
25-30	133(55.9)	105(44.1)	0.7(0.4,1.1))	
35-44	73(47.4))	81(52.6)	0.5(0.3,0.8)*	
>=45	73(64.6)	40(35.4))	1	
**Residence, no (%)**				*
Urban	108(50.9))	104(49.1)	1	
Rural	291(58.7)	205(41.3)	1.4(0.9,1.8)	

*Excluded in the final model

TB was considered a less stigmatized disease as compared to HIV/AIDS. On the other hand, a significant number of the FGD participants believe that TB and HIV have similar symptoms and people afraid of TB patients because of the associated HIV infection.

### Health seeking behavior and perception on the quality of service of the heath institution

A total of 49(6.5%) study participants had cough of 2 weeks or more during the survey and 23(46.9%) of them did not seek help in the health institutions or other places. Of those who had cough, 34.7% visited health institutions and 12.2% went to traditional healers. TB suspects in the study area usually did nothing for a long time or visited traditional healers to take herbal medicine like *Dama Kese *(*Qcimum sp*).

"*We usually drink or smell herbal medicine such as 'Dama Kese' for the treatment of **'Samba' (TB)**. If the disease becomes worse or not cured by the Dama Kese, we go to health facility*." A 28 years old woman at rural Kebele.

The majority of the FGD participants described that the quality of care in the nearby health posts or health centers is poor and people often do not visit them.

"*People would prefer health facilities for the treatment of **Samba**. However, the poor quality of service in the health posts and health centers forced people to take traditional drugs like Dama Kese*.", A 45 years old man in rural Kebele said.

## Discussion

The results of this study reveal several misconceptions and prejudice towards PLHA and patients with TB. More than 50% of the participants in this study do not know the possibility of transmission of HIV from a mother to child or by breast feeding. This figure is higher than previous reports from Ethiopia[38,39]. However, less than 10% of the study population believe that HIV can be transmitted by a mosquito/fly which is lower than the misconception reported in the 2005 Ethiopian Demographic Health Survey (40%) and the Ethiopian behavioral surveillance survey (20%) [38,39]. These differences could be attributable to several factors including the population being studied and the study periods. Our study primarily focuses on rural residents who might have different culture and views concerning TB and HIV as compared to urban and adolescent population[39].

The study reveals high stigmatizing attitude and the existence of real discrimination against PLHA in the community. A larger proportion of rural residents, females, non-literate individuals and those who have poor knowledge about HIV have high prejudice towards PLHA. This is consistent with the findings of a previous report in Ethiopia[38]. Inaccessibility of the HIV/AIDS interventions to the rural and disadvantaged segments of the population might explain the deep rooted prejudice in these communities. This prejudice can make PLHA reluctant to disclose their HIV status to their family [40,41]. PLHA may also suffer from verbal and physical abuse by the community and family members[42]. The low level of perception on the existence of HIV infection in the locality and the poor utilization of HIV/AIDS interventions such as condom and HIV counseling and testing will create a fertile ground for the rapid spread of HIV. On the other hand, the pervasive prejudice, discrimination of PLHA and low awareness concerning HIV/AIDS will be an important barrier for the HIV control program if it wants to expand ART to the grass root level and increase its uptake. Previous literatures had shown that low awareness and stigma were the major barriers for the free access of ART and other HIV/AIDS related cares [40,41,43]. The government of Ethiopia has launched Health Extension Program (HEP) to achieve the health related Millennium Development Goals (MDG)[44]. As part of the HEP, more than 30,000 health extension workers are trained. These health cadres are expected to provide a minimum preventive and promotive health packages to each household in a Kebele. HEP is a good opportunity for the TB/HIV control program to tackle the aforementioned challenge.

The majority (94%) of our study participants have heard of TB which is similar to other reports[33,34]. However, more than 50% of females and non-literate individuals do not know the cause of TB. Misconceptions concerning the cause of TB such as *cold air *and locally made brew[31] and lack of awareness on the major symptoms of TB can lead to a significant delay to seek help from health institutions. Less than 20% of the non-literates and females know ventilation and proper disposal of sputum as prevention methods of TB. Lack of awareness on the major modes of transmission of TB can be a potential hurdle for effective TB control in the community. The availability of two health extension workers in each Kebele can be a good opportunity to educate the rural community about TB.

The level of high prejudice towards TB in this study (56%) was lower compared to a report from China (89%)[33] and Thailand(65%)[45]. A higher proportion of females and non-literate individuals had prejudice towards a patient with TB which is consistent with other reports elsewhere[20,32,33,45]. The high level of public prejudice can affect the health care seeking behavior of TB patients[35,45]. Dissatisfaction of the community with the services of the health institutions was the major reason for not seeking help in the health institutions. The widespread prejudice, misconceptions, and the negative attitude of the community on the quality of care of the health institutions will be serious barriers for the TB control program to increase the low case detection rate(i.e. 27%) of pulmonary TB in the study area[3].

Despite the use of triangulation methods, the study has some limitations. First, the study was conducted in a predominantly rural population which might not be representative of the whole population of Ethiopia. Second, we did not do an assessment on the quality of care of the health institutions to triangulate the findings of the qualitative study. Third, we did not include TB or HIV patients to assess the real stigma and discrimination on patients' perspective.

## Conclusions

In conclusion, widespread prejudice (stigmatizing attitude) towards PLHA and patients with TB, misconceptions on the modes of transmission of HIV and TB and a negative perception of the quality of health services can be serious bottlenecks for the TB/HIV control programs to achieve the TB and HIV related millennium development goals (MDGs). TB/HIV control programs in collaboration with other partners should invest more in social mobilization and education of rural communities to rectify this. Enhanced HEP (by giving additional training for the HEW on TB and HIV) and outreach education campaign by trained health workers can be used as strategies to educate the rural community.

## Competing interests

The authors declare that they have no competing interests.

## Authors' contributions

**AD **was involved in the conception and design of the study, coordinated the field work, analyzed the data and drafted the manuscript. **GA **was involved in the conception, design of the study, field work and review of the article. **LA **was involved in the design and reviewed the article. **CJ**, **MT, JS, AA, KW, FD, MB, and AA **participated in the design, field work and reviewed the article. **RC **critically reviewed and approved the article. All authors read and approved the final manuscript.

## Pre-publication history

The pre-publication history for this paper can be accessed here:

http://www.biomedcentral.com/1471-2458/10/400/prepub
